# The effect of pre-hospital intubation on prognosis in infants, children and adolescents with severe traumatic brain injury: A protocol of systematic review and meta-analysis

**DOI:** 10.1097/MD.0000000000014690

**Published:** 2019-02-22

**Authors:** Yichen Guo, Ruilin Li

**Affiliations:** a Department of Emergency, The First Hospital of Lanzhou University; b Department of Neurology, The First Hospital of Lanzhou, Lanzhou, China.

**Keywords:** adolescent, children, mortality, pre-hospital intubation, prognosis, systematic review, traumatic brain injury

## Abstract

**Introduction:**

Traumatic brain injury is one of the leading causes of death and sources of heavy societal burden. Hypoxemia and hypercapnia are the 2 common complications of brain injury. Intubation seems to be an effective intervention for preventing the 2 complications in pre-hospital setting. But the results of the existing studies on the effect of pre-hospital intubation on prognosis of patients (aged less than 18) with severe traumatic brain injury are conflict. Thus, in this study, we aim to conduct a systematic review and meta-analysis to evaluate whether pre-hospital intubation is benefit for the prognosis in infants, children and adolescents with severe traumatic brain injury.

**Methods:**

We will develop a systematic search strategy which includes MEDLINE, EMBASE, Cochrane Central Register of Controlled Trials, Chinese Biomedical Literature Database, WanFang Data and China National Knowledge Infrastructure. The WHO International Clinical Trials Registry Platform will be searched for the ongoing studies as well. The cohort studies which aim to evaluate the effect of pre-hospital intubation for infants, children and adolescents with severe traumatic brain injury will be selected. The Newcastle-Ottawa Scale will be used for assessing the risk of bias of the included studies.

**Results:**

The results of this study will be presented in the full-text of the systematic review.

**Conclusion:**

This is the first systematic review and meta-analysis about evaluation of the effect of pre-hospital intubation on prognosis in infants, children and adolescents with traumatic brain injury.

**PRESPERO registration number:**

CRD42019121214

## 1 Introduction

Traumatic brain injury (TBI) is one of the leading causes of death and sources of heavy societal burden.^[1–3]^ There are many complications in patients with TBI, of which hypoxemia and hypercapnia are the most common. These two symptoms have been proved to associate with an increasing risk of mortality.^[4]^ Airway management like endotracheal intubation is commonly advocated for these symptoms in pre-hospital setting or hospital.^[5]^ However, the recommendation on pre-hospital intubation for patients with TBI is not supported by sufficient evidence, particularly in the guideline on infants, children and adolescents.^[6]^ At the meantime, the adherence to the recommendation seems to be commonly low in different countries.^[7,8]^ Although the existing systematic review suggests that prehospital intubation provided by non-well skilled physicians is related to higher risk of mortality compared to intubation in hospital, it hasn’t indicated that the action by well-skilled physicians doesn’t make sense.^[9]^

For the young patients aged < 18 years, they seem to have better prognosis theoretically than the adult patients due to young age. However, the current evidence is consistent. Some early studies showed that pre-hospital intubation had no significant advantage on survival rate or functional recovery,^[10,11]^ while a recent observational study suggested favorable long-term (6 months) effects by pre-hospital intubation compared to no intubation in pre-hospital setting.^[12]^ To date, there is no study systematically evaluating the effect of pre-hospital intubation on prognosis in infants, children and adolescents with TBI. In this systematic review, we aim to confirm whether pre-hospital intubation is effective on preventing young patients with TBI from death and improving their prognosis.

## 2 Methods

The protocol of this systematic review and meta-analysis has been registered in PROSPERO international prospective register of systematic reviews (register number: CRD42019121214. https://www.crd.york.ac.uk/PROSPERO/display_record.php?RecordID=121214). The protocol was designed in accordant with Cochrane Handbook for Systematic Reviews and reported according to the preferred reporting items for systematic review and meta-analysis protocol (PRISMA-P).^[13]^

### 2.1 Eligibility criteria

The studies will be included, if they meet the criteria below:

1. the patients with severe TBI,
2. age less than 18,
3. intend to compare the effect of pre-hospital intubation with no intubation,
4. cohort study,
5. report at least one of the outcomes: mortality (in hospital, three or six months after discharge), length of hospitalization, Glasgow Outcome Scale (GOS) (in hospital, or three or six months after discharge).

At early stage of study selection, the relevant systematic reviews will be included as well for tracking their references.

### 2.2 Information source

We plan to conduct a systematic search strategy which includes the following databases: MEDLINE (via PubMed), EMBASE, Cochrane Central Register of Controlled Trials, Chinese Biomedical Literature Database, WanFang Data and China National Knowledge Infrastructure. We also extend our search to the ongoing cohort study by searching the World Health Organization (WHO) International Clinical Trials Registry Platform (ICTRP) (http://apps.who.int/trialsearch/Default.aspx). In order to avoid missing the eligible studies, we plan to track the references of all the included studies finally.

### 2.3 Search strategy

We plan to perform the first electronic search from the inception of databases to December 31st 2018, and update the search before submitting the manuscript of this full systematic review to peer-review journal. The search terms related to TBI and intubation will be used. The search strategy below will be performed in PubMed:

1. #1 “Brain Injuries, Traumatic”[Mesh] OR “brain injury”[Title/Abstract] OR “brain injuries”[Title/Abstract] OR “head injury”[Title/Abstract] OR “head injuries”[Title/Abstract]
2. #2 “Intubation, Intratracheal”[Mesh] OR “prehospital intubation”[Title/Abstract] OR “pre-hospital intubation”[Title/Abstract]
3. #3 #1 AND #2

The details of search strategy can be found in the supplement file.

### 2.4 Study selection and data extraction

The references retrieved by electronic search will be imported to and managed by EndNote X7. Two independent reviewers will screen the references by checking the title and abstract firstly. And then, the full-texts of the potential eligible studies will be reviewed. We will design an electronic data form for our systematic review. The items we plan to extract from the primary studies include: title, first author, publication year, country, journal, source of funding, inclusion criteria of patients, records of intervention or exposure, sample size, age, diagnosis of patients in hospital, number of lost or withdrawal at the end of follow up, outcomes.

In order to have high inter-rater reliability between the independent reviewers, we plan to perform a pilot test for study selection and data extraction. When meeting disagreements, we will have a discussion on them or consult a third researcher to solve them.

### 2.5 Risk of bias assessment

The Newcastle-Ottawa Scale (NOS) for cohort study is still recommended for assessing the risk of bias of cohort study,^[14]^ even though a new risk of bias tool for non-randomized study has been developed.^[15]^ The NOS can well reflect the potential risk of bias of cohort study, which includes eight aspects (representativeness of the exposed cohort, selection of unexposed cohort, ascertainment of exposure, demonstration on that outcomes of interest was not present at the start of study, comparability of cohorts on the basis of the design or analysis controlled for confounders, assessment of outcome, long enough of the length of follow up to observe the outcomes, and adequacy of follow up of cohorts). Thus, the included studies in our systematic review will be evaluated by the NOS for cohort study. Eventually, the included studies will be evaluated as good, fair, and poor quality. When meeting disagreement, we will have a discussion or consult a third researcher.

### 2.6 Data synthesis

We plan to use STATA 12.0 to perform the meta-analysis. The dichotomous outcomes will be estimated by pooled risk ratio (RR) with 95% confidence interval (CI) and continuous outcomes by mean difference (MD) and 95% CI. *I*^*2*^ will be used for testing the heterogeneity between the studies. If *I*^*2*^ ≤ 50%, we will pool the data by Mantel-Haenszel fixed-effects model. Otherwise, sub-group analysis or meta-regression will be used to test the sources of heterogeneity. If the evidence of clinical heterogeneity is not apparent, the Mantel-Haenszel random-effects model will be used. If the heterogeneity is caused by clinical character and power is enough, we will perform sub-group analysis according to the clinical characteristics. But if the power is not enough, data synthesis will not be performed and a description of the results of the included studies will be given instead.

### 2.7 Quality of evidence

The quality of body of evidence will be assessed by Grading of Recommendation, Assessment, Development and Evaluation approach (GRADE) in the GRADEpro (GDT system: https://gradepro.org/).^[16,17]^ Quality of evidence is an important aspect for the guideline developers to formulate a recommendation and for the other evidence end-users such as doctor, patient or researcher to understand how much confidence the results can be believed. The evaluation of quality of evidence in systematic review is likely to benefit the dissemination of the evidence. The quality of evidence can be rated by GRADE as high, moderate, low and very low. The following five factors will be considered for downgraded the quality of evidence: risk of bias, directness, inconsistency, imprecision of effect estimates and publication bias. The quality of body of evidence from observational studies is set as low primarily. If the quality of evidence from cohort studies hasn’t been downgraded by the above five factors, we will further consider whether it can be upgraded by the three factors: large magnitude of effect, dose-response gradient and plausible confounding.

### 2.8 Ethics and dissemination

This study is a systematic review and meta-analysis. So, there is no requirement of ethical approval and patient informed consent.

## 3 Discussion

There are 2 previous systematic reviews on this topic.^[9,18]^ One was published in 2009, but it couldn’t conclude a conclusion whether pre-hospital intubation was effective for the limited evidence at that time.^[9]^ Another one was published in 2015, but only focused on the adult patients.^[18]^ There are several studies on this area published after the two systematic reviews.^[12,19]^ Thus, in this systematic review, the evidence on the effect of pre-hospital intubation for patients (aged < 18) with severe TBI will be firstly comprehensively evaluated.

## Author contributions

Conception and design of this systematic review and meta-analysis (Yichen Guo, Ruilin Li); tested the feasibility of the study (Yichen Guo); developed the search strategy (Yichen Guo); drafted this protocol (Yichen Guo), revised the protocol (Yichen Guo, Ruilin Li). All authors provided critical revisions of the protocol and approved the final manuscript.

**Conceptualization:** Yichen Guo, Ruilin Li.

**Investigation:** Yichen Guo, Ruilin Li.

**Methodology:** Yichen Guo.

**Resources:** Yichen Guo, Ruilin Li.

**Software:** Yichen Guo.

**Validation:** Yichen Guo, Ruilin Li.

**Writing – original draft:** Yichen Guo.
